# How to collaborate for health throughout the project timeline – a longitudinal study reflecting on implemented strategies in three projects for a healthy living environment

**DOI:** 10.1186/s12889-022-14898-9

**Published:** 2023-01-10

**Authors:** N. J. E. van Vooren, L. M. S. Janssen, H. W. Drewes, C. A. Baan, I. M. B. Bongers

**Affiliations:** 1 Department of Quality of Care and Health Economics, National Institute for Public Health and the Environment (RIVM), Centre for Nutrition, Prevention and Health Services, P.O. Box 1, 3720 BA Bilthoven, The Netherlands; 2 Tilburg University, Tranzo, Tilburg School of Social and Behavioural Sciences, PO Box 90153, Tilburg, 5000 LE The Netherlands; 3 Mental Health Care Institute Eindhoven, de Kempen, PO Box 909, Eindhoven, 5600 AX The Netherlands

**Keywords:** Cross-sector collaboration, Healthy living environment, Realist evaluation

## Abstract

**Background:**

When improving the health of local and regional populations, cross-sector collaboration between different policy domains, non-governmental organisations and citizens themselves is needed. Previously, enabling factors and strategies have been identified to improve cross-sector collaboration for health. However, few longitudinal studies have been conducted to understand how the implementation of strategies for cross-sector collaboration changes throughout the collaboration process. The aim of this study is therefore to learn more about the different strategies that were implemented throughout three cross-sector collaboration projects for a healthy living environment.

**Methods:**

The realist evaluation approach was used to understand how the implemented strategies worked, in which context, why and with what outcomes. Project partners were asked to reflect on their implemented strategies at two different moments in the project timelines, and quarterly updates with project leaders were held. In addition two reference panels were organised for data triangulation.

**Results:**

Three key insights for successful cross-sector collaboration throughout projects for a healthy living environment were identified, namely 1. Investing in trust among the partners and faith in the project has a positive influence on continuing the collaboration throughout the project; 2. Making stakeholders actively participate throughout the project requires additional strategies after the onset of the project, and 3. Defining roles, tasks, and other prerequisites at the start of the project helps in pursuing the project over time, but needs re-examination throughout the project. These key insights were based on multiple examples of implemented strategies, linked to context, mechanisms and outcomes.

**Conclusions:**

This study shows the different strategies that can be employed as the collaboration in projects for a healthy living environment progresses. We found that ‘trust’ does not merely include the relationships built between the partners, but at the onset of projects can also be based on faith in the project itself. In addition, as it can be difficult to foresee the right investments and strategies at the onset of the project, frequent reflection moments to choose fitting strategies might benefit regional partners in their cross-sector collaboration for health.

**Supplementary Information:**

The online version contains supplementary material available at 10.1186/s12889-022-14898-9.

## Background

Over the years we have learned that health is not only affected by personal factors or lifestyle, but also by environmental factors, both physical and social [1, 2]. Multiple studies show the relation between the physical environment and health. For example, the built environment can be used to promote physical activity [3] and living in a green environment was found positive for health [4, 5]. This means that addressing the health of local and regional populations does not merely concern the public health or health sectors alone, but also the social domain, environmental domain and other sectors are involved [1]. Multiple approaches and initiatives have been developed to promote collaboration between different policy sectors, institutes and citizens, aimed at improving the health of their local and regional populations [6–8]. An example is the Health in all Policies approach, which aims to include health in policy decision-making across sectors [9–11]. Furthermore, numerous thematically focused cross-sector collaboration initiatives have been developed, focusing on topics like childhood obesity, improving lifestyle, mental health or a healthy living environment [12, 13].

Cross-sector collaboration is based on the idea that multiple sectors share resources, information and activities in order to achieve an outcome, like improved health for a certain population, that could not be achieved by the organizations in one sector alone [14]. This collaboration has been studied widely, providing insights in preconditions for collaboration, enablers, barriers and the link between them [11, 12, 15–19]. A common understanding in these studies is that successful cross-sector collaboration cannot simply be achieved by addressing a list of enablers or applying certain strategies. Instead, the effectiveness of enablers and strategies is influenced by different and changing contexts throughout the collaboration process [14, 20, 21]. An increasing number of realist studies have described how different contexts affect the outcomes of collaborative strategies [22, 23]. These studies helped our understanding of how to act upon the complexity of collaboration by learning which strategies and factors to apply, how they work in different contexts, why and with what outcomes.

Even though these studies provide an increasing understanding of how to act upon the complexity of cross-sector collaboration, they provide little insight in how strategies might change during the collaboration process. Several scholars have identified a different use of strategies at different moments during the collaboration process [9, 24, 25]. Scholars therefore advocate for studies with a more longitudinal approach to better understand cross-sector collaboration [15]. However, most studies in cross-sector collaboration are based on overall lessons of case studies or on cross-sectional studies [12, 15]. In order to gain insight in how strategies for cross-sector collaboration change over time, this study therefore applied a more longitudinal approach among three projects that focused on creating a healthy living environment. The following research questions will be answered:

- 1. Which strategies aimed at improving cross-sector collaboration were implemented throughout projects for a healthy living environment, in which contexts, triggering which mechanisms and to which outcomes?
- 2. What are the key insights when comparing the implemented strategies throughout the projects?

## Methods

### Setting

This study is part of a research project running from 2019–2022 which was initiated by the Dutch National Institute for Health and the Environment (RIVM). Aim of the project is to learn how to improve collaboration with local and regional partners when working on a healthy living environment. Being part of this project, the current study focuses on the experiences within three collaborative projects in three different provinces of the Netherlands (see Table 1 for more detailed information about the three projects). This selection of projects was based on the fact that 1) they address a variety of policymaking themes aimed at creating a healthy living environment, and 2) at the onset of the projects there was a need for cross-sector collaboration to address the aim of the projects.

**Table 1:** Description of the three projects included in this study

Project A: Local policy priority setting with citizens. This project focuses on implementing a method for involving citizens in local policy priority setting for a healthy living environment. The project started in 2018 within one Dutch province, in which the local priority setting was performed with several citizen groups. Currently (January 2022), follow-up activities based on this priority setting are being implemented. The experiences of collaboration from start until January 2022 are subject of this study. Most of the partners participating within this province are included in the ‘daily board’ of the project, in which the course of the project is discussed. These partners are RIVM, universities of applied science, regional public health services, regional safety services and the participating municipality within this province. This daily board is embedded within a larger consortium which includes similar partners from two other provinces. Within this consortium, knowledge is shared across the provinces
Project B: Understanding the influence of large scale farming on air quality by measuring air quality with famers and citizens. In the local (rural) area of this project, citizens and farmers had different perspectives on the influence of large scale farming on the local air quality. In 2019, together with the municipality, province and RIVM a project was started for them to measure the air quality together. The aim of this project was twofold; 1. create insight in the local air quality and 2. facilitate the local discussion for follow-up actions, by creating trust amongst the local partners during the project. The ‘core group’ within this project included representatives of both citizens and farmers, representatives of the municipality and the province, and RIVM, who provided the project leader for the project. In this core group, decisions were made about the course of the project. Additionally, citizens and farmers were included in the project to perform the measurements for the air quality. By the end of 2021 results of measurements were discussed and a dialogue between citizens and farmers about solutions for cleaner air was held. The partners presented lessons learned to the broader local and regional community
Project C: Creating a knowledge base to assist local policy decisions regarding the effect of climate change on health. This project was based on a continuation of an existing consortium preparing a research project for a research call. As the original research call didn’t result in a grant for the research project the lead partners of the consortium started another research project in 2019 together with the RIVM. The new research project focused on creating a knowledge base about the effects of climate adaptation and mitigation on health to facilitate local and regional decision making. The ‘core group’ within this project included a University of applied science, province and the regional public health service. Partners involved were apart from the core group other municipality’s and waterboards. The results of the project with RIVM were based on a needs assessment and literature review, and were presented to the local and regional partners in 2021

### Realist evaluation approach

In this study the Realist Evaluation approach was used to gain better insight in how strategies lead to certain outcomes for cross-sector collaboration, and how this is affected by context. The Realist Evaluation approach acknowledges the idea that observational evidence alone cannot explain the causal relations between strategies, contexts and outcomes. Insight into why this relation is formed, namely the mechanisms that are triggered, is needed [26]. By using the heuristic of Context-Mechanism-Outcome configurations (CMO’s), relations between how contextual factors trigger certain mechanisms and lead to specific outcomes are searched [27]. This study explicitly adds the implemented strategies to the CMO configurations, to be able to form action-oriented insights in how to collaborate across sectors [28]. The definitions of the Strategies, Contexts, Mechanisms and Outcomes that are used in this study are described in Table 2.

**Table 2:** Conceptualizations of S-C-M–O (copied from van Vooren et al. (2020) [29])

Strategy	Refers to intended plans of action [27]. In this study the strategies are aimed at achieving cross-sector collaboration for a healthy living environment
Context	Pertains to the ‘backdrop’ of programs, which can be understood as any condition that triggers or modifies the mechanism [27]. In this study, the contextual conditions can be the different multilevel sociocultural, relational, economic, political or historical conditions in which the strategies are implemented, which in turn causes certain mechanisms to be triggered
Mechanism	Refers to the generative force that leads to outcomes [27]. Mechanisms should not be mistaken for strategies, as strategies are seen as intended plans of action, whereas mechanisms are the responses to the intentional resources that are offered [27]
Outcome	Refers to the intended or unintended process outcomes [27]. This study focuses on the outcomes of strategies for achieving cross-sector collaboration for a healthy living environment

### Theoretical framework

**Fig. 1:**
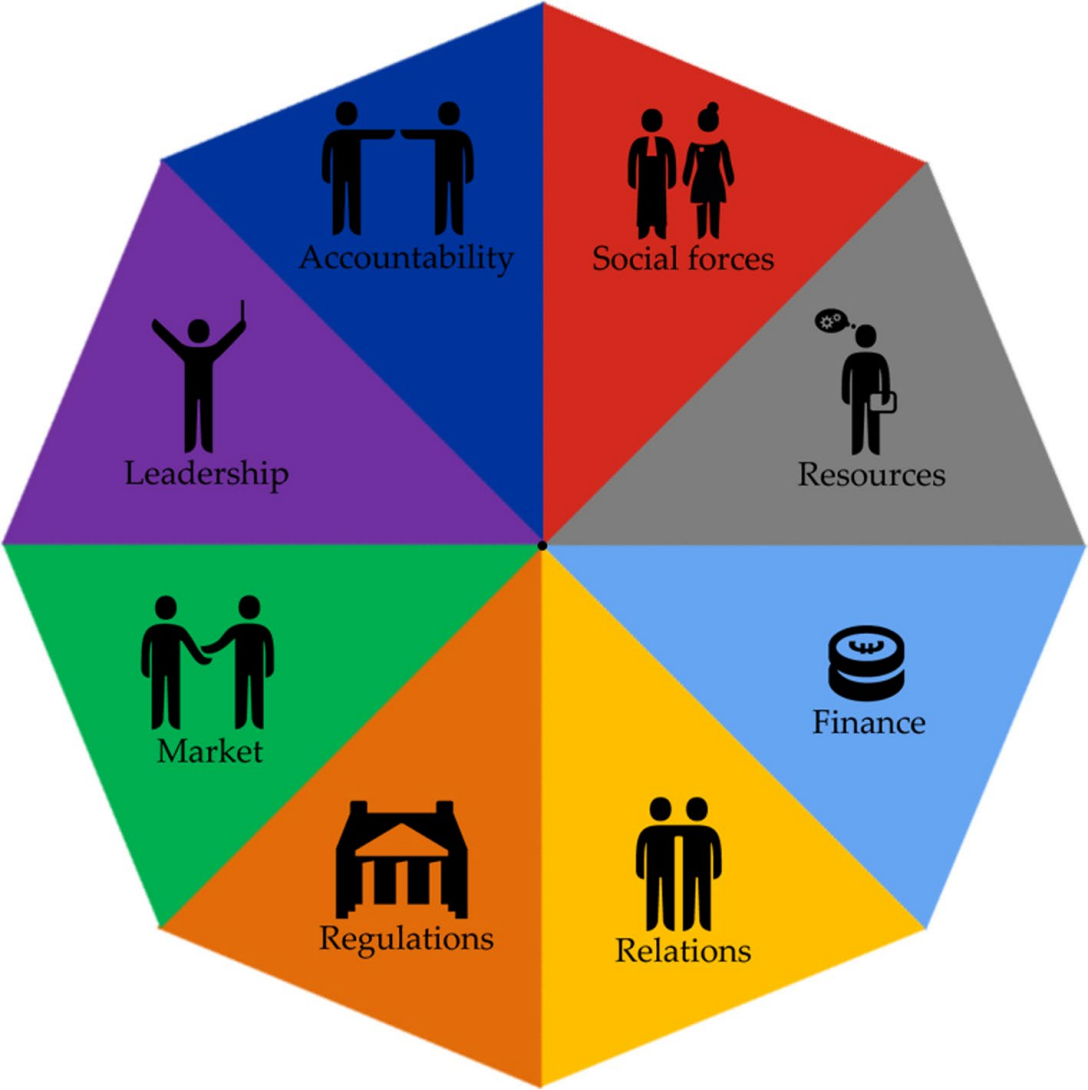
Visualization of the eight CAHN components (copied from van Vooren et al. (2020) [29]

The Collaborative Adaptive Health Network (CAHN) framework is used to help understand the factors that influence the process of collaboration. The CAHN framework is based on an international realist synthesis including literature focused on collaboration between different sectors (healthcare, social care, community services, public health) [30]. The framework includes eight components affecting cross-sector collaboration for population health management. These components are further divided in 38 subcomponents. This framework has been used before in studies aiming to understand the collaboration across sectors for population health management, and collaboration for a healthy living environment [29, 30]. See Fig. 1 for the visualization of the eight CAHN components.

### Data collection

To learn how the implementation of strategies might change throughout the project timeline, three data sources were used for data triangulation: 1) The project partners of the three projects were asked to reflect on their implemented strategies in semi-structured interviews at two moments during their collaboration project, 2) quarterly updates with project leaders were held throughout the project timelines, 3) and also two reference panels were asked to reflect on the implemented strategies throughout the three projects. The data collection with these three sources will be discussed in more detail below.

Semi-structured interviews to reflect on the implemented strategies in the projects were held at two moments throughout the project timelines. The first reflection moment focused on implemented strategies from the onset of the collaboration, and included 17 interviews with the partners that were mainly involved in starting up the projects.The results of this first reflection moment are described in van Vooren, Drewes [29]. This study will add to these insights by, amongst others, comparing the findings of a second reflection moment two years later (see Table 1 for the status of the projects during the second reflection moment), with the implemented strategies after the onset of the collaboration. For the second reflection moment, the sample strategy aimed to include as much as possible the same partners that were interviewed about their experiences before, as they would be able to reflect on their experiences throughout the total project timeline. Fourteen partners were interviewed, which included five partners from project A, seven partners from project B, and two partners from project C. Thirteen of those partners were interviewed before, and thus had experienced the collaboration throughout the total timeline of the project. Due to personnel changes one new representative within project B was interviewed. The interviewed partners of the three projects consisted of researchers from RIVM (3), representatives of municipalities (3), representatives of provinces (regional governments) (2), farmer representatives (2), citizen representatives (1), a representative of a university of applied science (1), a representative of the regional safety services (1), and a representative of the regional public health services (1). See Additional file 1 for the interviewed partners per project.

The semi-structured interviews were based on an interview guide that was mostly similar to the first reflection moment, in order to be able to compare the implemented strategies. The CAHN framework was used consequently throughout all interviews to maintain a broad and theory-based perspective throughout the interview. The partners were asked about their objectives and aims with the collaboration and the projects (and whether this changed over the two years). Furthermore, partners were asked about their experiences regarding implemented strategies in the collaboration in the past two years, what worked (or not), how and why. After starting with broad questions about their experiences and implemented strategies, a visual representation of the CAHN framework was shown and explained to all partners to provide them with the possibility to reflect on additional experiences when applicable (in case they were not mentioned before). The interviews had a duration of about one hour and were all performed via video-call due to restrictions during the COVID-19 pandemic.

In addition to the semi-structured interviews, from October 2019 until January 2021 quarterly updates were held with project leaders of the cases, working at the RIVM, which served to get updates about possible changes in context throughout the projects.

To improve external validity and be able to reflect upon the experiences from additional cross-sector collaboration contexts the results of the interviews were discussed with two reference panels. One reference panel consisted of 12 researchers from the RIVM who had experience working in these, and other regional collaborative projects, or who had an assignment in arranging the organizational prerequisites for regional collaboration. The other reference panel consisted of 10 researchers from Tranzo at Tilburg University, all with experience with regional collaboration in research projects.

### Analysis

Informed consent was asked for recording and using the data of the interviews. The research process and the informed consent forms were approved by the Ethical Review Board of Tilburg University (EC-2019.75). The recordings of the interviews were transcribed literally and where analyzed in MaxQDA 2020. Two researchers (NvV and LJ) coded the interviews based on the Realist evaluation approach. This means that within the interviews, causal links between strategies, contexts, mechanisms, and outcomes were searched. The SCMO configurations were coded within the CAHN framework by fitting the mechanisms within the 38 subcomponents of the CAHN framework. The two researchers started with each coding the same three interviews, in order to check for inter coder agreement. After these three interviews, the other interviews were divided and coded by one researcher and cross-checked by the other. Whenever there was disagreement of codes, there was another cross-check by the researchers whether and how to include this code in the eventual analysis. In addition to these SCMO configurations, the notes of the quarterly updates with the project leaders provided further contextual insights, helping in clarifying SCMO’s or adding insights for new SCMO’s.

After coding all configurations, these configurations were compared with the insights from the first interview round. To do this, the configurations of the second interview round were clustered within the seven themes and underlying strategies that were found in the first round (see Additional file 2 for the seven identified themes for cross-sector collaboration). One researcher (NvV) compared whether experiences after two years were still applicable to the previous findings, and whether different strategies were used, which in turn was checked by the other members of the research team (LJ, IB, CB, HW). Based on this comparison, overall insights for collaboration throughout the project timelines were formulated. These overall insights were discussed in the two reference panels to further validate them in a broader context.

## Results

Comparing the SCMO configurations over the years provided three key insights for successful collaboration throughout the projects. These are: 1. Investing in trust among the partners and trust in the project has a positive influence on commitment throughout the project; 2. Making stakeholders actively participate throughout the collaborative project requires additional strategies after the onset of the collaboration project; 3. Defining roles, tasks, and other prerequisites at the start of the project helps in pursuing the project over time, but requires re-examination throughout the project. An explanation of how the partners of the projects have addressed these insights is described below. See Additional file 3 for more examples of the Strategy (S) – Context (C)– Mechanism (M) – Outcome (O) configurations that formed the basis of these overall insights.

### Investing in trust among the partners and faith in the project has a positive influence on continuing the collaboration throughout the project.

The importance of investing in trust for continuing the collaboration was mentioned in both reflection rounds and the project updates, and it was also recognized as important by the reference panels. Depending on the context of the collaboration, different strategies were implemented to address different types of trust.

For example, when reflecting upon the collaboration process of two of the projects, investing in the relationships among partners from the onset of the project and onwards (S), was mentioned as facilitating for overcoming complexities throughout the project (O). These complexities were related to changes of context or differences in needs among partners (e.g. dealing with the corona pandemic as a priority, or dealing with local pressure of residents) (C). As the partners of the project invested in their relationships, they were able to create openness and trust among each other. This openness helped to clear the air when needed, and helped build a base to discuss everyone’s tasks (M).

“The mutual trust which was built at the start, was of such a level that […] we trusted this [external local] conflict would not have an effect within this group” (R2_Interview9)

However, when starting a project with a lack of trust among several partners, as was the case in project B, investing in faith in the project itself seemed important for partners’ commitment to the collaboration. A strategy for creating faith within the project itself was including an independent and neutral project leader, who provided the other partners with the ability to be open about their different perspectives, and could steer the discussion towards the mutual objectives and research facts when needed (S). This project leader gave partners with different perspectives on the same topic (C) the trust throughout the project that the discussion and project would be handled correctly and fairly. This helped in overcoming different perspectives (M), and was found facilitating for pursuing the collaboration in the project (O). Other examples of strategies used to create trust are described in Additional file 3.

“When you talk about [name of project] then […] there is a risk of distrust among each other. One says this, the other says that it isn’t correct. Discussions will take place. But when you have an independent person, who can say, these are the facts, and we have measured this, with scientific foundation, then you talk about the facts. And from there you can continue the conversation, how do we interpret these facts” (R2_Interview 12)

### Making stakeholders actively participate throughout the collaborative project requires additional strategies after the onset of the collaboration project

When reflecting on the starting phases of the projects, partners implemented a lot of strategies that were aimed at including and maintaining stakeholders at the onset of the collaboration. Strategies were, for example, related to creating a feeling of urgency, and creating a positive trade-off between the objectives of the project versus the organizational objectives. These were strategies that were also mentioned to be important later in the project.

Additionally, further in the projects we found that stakeholders, after agreeing to participate at the onset of the project, did not always actively participate and show ownership as the project progressed. The lack of active participation became problematic at the moments in the projects when concrete actions were needed from the partners (e.g. local partners like municipalities were needed in the implementation phase). Multiple strategies for addressing active participation were mentioned, of which several examples are described below (See Additional file 3 for more examples).

Learning about, and addressing the needs and priorities of local and regional project partners (S) was a strategy that was reflected on by the partners of two projects in which both national and local partners collaborated. Learning about the context of local partners helped the RIVM for instance to better align the project to the local needs. This alignment was found necessary when commitment at the onset of projects was needed. It also seemed necessary later in the project, in a situation where active participation was needed from partners, but where there were no other mechanisms like financial incentives or loci of accountability to achieve this participation (C). In this situation one is dependent on the partners’ trade-off of organizational needs vs. project needs, for their choice of active participation (M), influencing the possible output of the collaborative project (O).

“Of course, different partners of the consortium can invest in [name of project], but eventually it is up to the municipality if it can continue or not.” (R2_Interview3)

Another strategy that influenced partners’ levels of participation was based on the type of leadership that was used during the project (S). In the projects, due to different circumstances, the RIVM took a leading role in (part of) the project (C). In two of the projects, the project leaders experienced difficulty in sharing ownership and tasks with other project partners during their collaboration (O). It was reflected in one project that taking too much of a leading role might have influenced the participation of other partners (M). An experience in another project was that taking a leading role and being not sufficiently aligned to the needs and priorities of the other partners resulted in the project being experienced as the RIVM’s project and not one ‘of the partners’(M). The project partners did not always actively participate, as it was not always felt as if it was ‘their project’. In the third project there was a focus on shared leadership, which made partners discuss tasks and situations together and which was appreciated by multiple partners.

“And then you can say, we want to retrieve information to the, for the region itself, but eventually you will retrieve more information for the RIVM […]. And I can imagine that for the region [means regional partners] it was less interesting to take part in it [the project]. Or that they had the feeling: this is a thing of the institute, they will get there themselves.” (R2_Interview1)

In two projects continuous participation of local residents was needed during the execution stages of the projects. A strategy used in one project included having a good representation of the residents in the project core group (S). In the context where the duration of research projects was found not to be in sync with residents’ needs for results, and where there was an extra delay due to the Covid-19 pandemic (C), having a resident representative in the core group whom translated the questions and discussions between residents and the ‘project’, helped to keep the residents motivated and committed to participate in the project (M). This resulted in a continued commitment of most residents, despite the delay of the project (O).

### Defining roles, tasks, and other prerequisites at the start of the project helps in pursuing the project over time, but requires re-examination throughout the project

Several strategies were mentioned that were related to project management, namely in defining roles, tasks and other prerequisites for the project. Throughout the projects, partners from all three projects reflected on strategies related to division of roles and tasks. In the later phases of the projects the partners also reflected on the need to predefine and address the necessary expertise and other prerequisites (e.g. finances and accountability) at the start of the project, as this was found to be a constraining factor for the progress of the project. When discussing with the reference panels however, it was mentioned that it might be difficult to foresee which resources, roles and tasks are needed throughout the entire course of the project. Also they mentioned that sometimes a deliberate decision was made to start small and act first, whereby they only learned throughout the project what additional resources were needed. An example about how partners addressed the role and task division is described below (see Additional file 3 for more examples).

The strategy to make agreements about everyone’s roles, and tasks (S) was already acted upon from the start of the projects, and also two years later this strategy appeared a necessity. Right after starting the project, these agreements were needed for everyone to know what was expected of them. However when collaborating throughout the years with partners from different sectors (C), the basis of mutual agreements and investment in getting to know each other (S), not only created clarity of task division, but also provided a safe basis for partners to call on each other whether tasks where performed or not (M). This helped in addressing the necessary input of each partner for further collaboration in the project (O).

“I think it is useful to describe the tasks and roles at the start of the project. It is valuable. You see that especially eh.. in the grey area, there is discussion. For example when the workload increases and concrete actions are needed. Well, is this for organization A or B? […] And having built some relationships helps in having this discussion [….] it makes it easier to talk about, and less intimidating, or with les tension compared to when there is more relational distance between each other.” (R2_Interview3)

The changing needs of roles and expertise was explicitly mentioned in one of the projects, showing the need to link the right task to the right person (whom is also accountable for it) (S). In this example a partner representative was involved in the project for certain tasks defined at the start of the project. During the project, the tasks for the projects shifted and these were no longer within the original expertise and agreed upon responsibility of the representative (C). This resulted in a deliberation of how the representative would participate on this task (especially with a lack of hours to spend on these tasks) (M). There was a disbalance between the person that was included in the collaboration, and the tasks and responsibilities that were asked throughout the project (O).

## Discussion

The aim of this study was to learn more about the different strategies that were used for cross-sector collaboration throughout the timeline of a project. Three key insights for collaborating throughout the timeline of the project were formulated. The use of the realist evaluation approach provided more detailed insight into how these three overall insights could be addressed, in which context, why and to which outcomes. Even though several studies have pointed out the possible difference in strategies that are implemented throughout the process of collaboration [24, 25], by our knowledge this study is the first in which the use of strategies is followed throughout projects for a healthy living environment [12, 15].

The themes related to the three key insights (trust, commitment, clarity of roles, tasks and prerequisites) were found to be important in previous literature as well [11, 12, 15, 16]. These studies had already discussed that needs pertaining to these themes might change as collaboration progresses [12, 15, 16]. Our study adds to the understanding of how and why different strategies can be employed as the project progresses, in order to address these changing needs. For example in order to achieve commitment or active participation, or to effectively deal with different situations of trust within a project.

Furthermore, relating to the first key insight in the results *‘Investing in trust among the partners and faith in the project has a positive influence on proceeding the collaboration throughout the project’* this study identified the necessity of explicitly defining what is meant by strategies related to ‘trust building’. The building of trust is a commonly named strategy for effective cross-sector collaboration [15, 16, 31]. In most of these studies this strategy is related to building relationships between the partners. However, as was found both in literature and in our study, building relationships takes time and mostly develops throughout the duration of the project [12]. Thus ‘trust building’ based on relationships between the partners might not yet be sufficient at the onset of the project. This study provided first insights in the importance of strategies related to ‘creating faith in the project’ for trust building (e.g. when no trusting relationships exist yet, faith in the fairness of the project was valued by having an independent project leader). This adds to the understanding that investing in ‘trust building’ is not merely about investing in relationships among partners, but it is also about faith in the project itself.

In key insight three, the interviewees in our study mentioned several strategies that ideally should have been implemented at the start of the project to facilitate the collaboration. At the same time, the reflection panel in our study discussed that partners might not be able to foresee what is needed throughout the project, and that needs can change during the project, thus adaptation moments are needed. More studies have described the complexity of collaboration due to its continuously changing contexts [9, 12]. In literature one of the ways to address the needs pertaining this dynamic context is to include a continuous learning process [14, 18, 20, 25]. Suggested both in our study and by literature, frequent reflection moments can provide moments to adjust the strategies for collaboration throughout the project whenever needed. During these reflection moments, the insights from this study can aid in helping project leaders choose which strategies to implement at which moment in the project.

This study included three projects that all had a different focus for creating a healthy living environment. By choosing a variety of projects (of which project C even experienced diminishing collaboration throughout the timeline) and by validating them with reference panels that have experience in other contexts as well, this study tried to increase the generalizability of its findings. However, as in other qualitative studies in which a limited amount of cases could be followed, in order to further validate the findings of this study in other collaboration contexts, additional longitudinal studies in different cross-sectional collaboration contexts will be valuable.

## Conclusions

This study aimed to learn about the key insights for collaborating throughout a project for a healthy living environment based on strategies that were used in three projects. For improving cross-sector collaboration throughout the project timeline, key insights show that investing in trust, commitment and clarity of roles, tasks and prerequisites is needed. This study shows the different strategies that can be employed in order to properly address these themes as the collaboration in projects progresses, and how the use of strategies in a certain contexts triggers mechanisms and affects outcomes of collaboration. When comparing strategies throughout the timeline, we found that investing in ‘trust’ does not merely have to include the relationships built between the partners, but at the onset of projects can also be based on faith in the project itself. In addition, it was found necessary to timely address prerequisites for the continuation of the collaboration, though these can be difficult to foresee at the onset of the project. Thereforefrequent reflection moments to choose fitting strategies might benefit regional partners in their collaboration.

## Supplementary Information

**Additional file 1. **Interviewed partners per project.

**Additional file 2. **Themes for addressing cross-sector collaboration according to van Vooren et al. (2020).

**Additional file 3.** Examples of Strategy-Context-Mechanism-Outcome configurations for the three overall insights.

## Data Availability

The data sets generated during the analysis of the current study are included in the additional files of the article. Any templates used for data collection and analysis are available from the corresponding author on reasonable request.

## Abbreviations

CAHN: Collaborative Adaptive Health Network framework
SCMO: Strategy-context-mechanism-outcome configuration
RIVM: National institute for public health and the environment (Dutch: Rijksinstituut voor Volksgezondheid en Milieu)
