# Supplementary material for: How to collaborate for health throughout the project timeline – a longitudinal study reflecting on implemented strategies in three projects for a healthy living environment
Source: BMC Public Health. 2023 Jan 10;23:67. doi: 10.1186/s12889-022-14898-9 (PMC9831012; doi:10.1186/s12889-022-14898-9)
Supplement: Supplementary file 1 — Additional file 1. Interviewed partners per project. [file 12889_2022_14898_MOESM1_ESM.docx]

# Additional file 1. Interviewed partners per project

|  | **Project A** | **Project B** | **Project C** |
| --- | --- | --- | --- |
| Municipality | 1 | 2 | N/A |
| Province | N/A | 1 | 1 |
| Citizen representative | N/A | 1 | N/A |
| Farmer representative | N/A | 2 | N/A |
| University of applied science | 1 | N/A | N/A |
| RIVM | 1 | 1 | 1 |
| Regional safety services | 1 | N/A | N/A |
| Regional public health services | 1 | N/A | -* |
| **Total** | **5** | **7** | **2** |

N/A: not applicable for this project (these type of partners were not substantively included in the collaboration in this project)

*The representative of the regional public health services did not participate in an interview, as they mentioned that due to the diminished collaboration, no additional experiences occurred.
