# Supplementary material for: How to collaborate for health throughout the project timeline – a longitudinal study reflecting on implemented strategies in three projects for a healthy living environment
Source: BMC Public Health. 2023 Jan 10;23:67. doi: 10.1186/s12889-022-14898-9 (PMC9831012; doi:10.1186/s12889-022-14898-9)
Supplement: Supplementary file 2 — Additional file 2. Themes for addressing cross-sector collaboration according to van Vooren et al. (2020). [file 12889_2022_14898_MOESM2_ESM.docx]

# Additional file 2. Themes for addressing cross-sector collaboration according to van Vooren et al (2020b)

1. Creating a feeling of equivalence among the partners
2. Building trust among the partners
3. Creating a connection between the different sectors and perspectives
4. Providing clarity about roles and tasks
5. Creating and leveraging reasons to commit to the cross-sector project
6. Making sure the partners feel engaged within the cross-sector project
7. Understanding whom to engage at which point of the process.
