# Supplementary material for: How to collaborate for health throughout the project timeline – a longitudinal study reflecting on implemented strategies in three projects for a healthy living environment
Source: BMC Public Health. 2023 Jan 10;23:67. doi: 10.1186/s12889-022-14898-9 (PMC9831012; doi:10.1186/s12889-022-14898-9)
Supplement: Supplementary file 3 — Additional file 3. Examples of Strategy-Context-Mechanism-Outcome configurations for the three overall insights. [file 12889_2022_14898_MOESM3_ESM.docx]

# Additional file 3. Examples of Strategy-Context-Mechanism-Outcome configurations for the three overall insights

**1. Investing in trust among the partners and faith in the project has a positive influence on continuing the collaboration throughout the project**

| **Strategy** | **Context** | **Mechanism** | **Outcome** | **CAHN** |
| --- | --- | --- | --- | --- |
| Having an independent project leader | A rural area where partners (e.g. farmers and citizens) had contradictory perspectives about the topic of the project. However, there was a communal interest to address this topic. The national knowledge institute was asked to be the project leader, as they were perceived independent and with expertise on the topic. | 1. Having an independent project leader that was focused on the ‘facts’ surrounding the issue, helped discussions to focus on these facts, rather than each partners’ own perspective. The partners were able to express their own perspectives and accept their differences.  2. As there was an independent project leader, the other partners felt able to express their own perspective during collaborative meetings. They didn’t have to be neutral or participate with different ‘hats’ on. | 1. This made the partners trust the research in the project (as it was not influenced by subjective perspectives).  2. This helped the partner to express himself during the collaboration. | Leadership - representation |
| The project leader aims for a form of shared leadership | A context with an independent project leader and partners with different perspectives. The project leader aims for inclusive leadership, by providing each partner the possibility to speak and share ideas. | This type of leadership created a culture in which discussions were addressed collectively. Only after a collective decision was made about an issue, action was taken. | Partners from this project applauded this type of leadership. Another partner spoke about the importance of trust that was created amongst the group. | Leadership - commited leadership |
| Investing in relationships from the start of the project | A rural context in which the issue at hand became increasingly intertwined with a larger societal debate during the course of the project | As the partners of the project invested in their collaboration before the influence of the societal debate, they were able to create openness and trust among each other. This openness helped clearing the air when needed, e.g. in moments that external factors influenced the discussions of the partners within the project. | This prevented the external societal debate to influence the collaboration within the project (though more local issues were sometimes addressed within the project). | Market - trust reciprocity - respect |

**2. Making stakeholders actively participate throughout the project requires additional strategies after the onset of the project**

| **Strategy** | **Context** | **Mechanism** | **Outcome** | **CAHN** |
| --- | --- | --- | --- | --- |
| Learning about, and addressing the needs and priorities of local and regional project partners  *This was found to be more difficult when physical visits and face-to-face contacts were inhibited during the corona pandemic* | A research pilot for a healthy living environment, with a.o. the involvement of municipalities. These municipalities experience shortages in budget and resources and need to prioritize. The timing of these interviews was during the corona pandemic and  municipal elections, influencing the prioritization process. | 1. As the project is a pilot, this can be perceived as ‘extra’. Which not always matches the other priorities in the municipality.  2. The municipal elections have an influence on the prioritization of the alderman  3. Because the theme ‘healthy living environment’ has not yet been allocated to a certain municipal domain, and people are not yet held accountable for this theme, it has not yet become a priority. | 1 and 2. These mechanisms were experienced (by other partners) as the possible reasons for municipalities to hold back on active participation.  3. The lack of accountability is found to be an inhibiting factor in the level of participation in the project | 1. Market - engagement  2. Regulations - political influence  3. Accountability – incentive design |
| Use financial incentives to create accountability (hypothesis) | A research pilot for a healthy living environment, where the participation of partners is dependent on their experienced urgency to participate | Active participation depended on how partners balanced the project needs with their own organizational needs. The need for an incentive design for accountability, to be able to steer for active participation, was felt by the project leader | It was hypothesized that when having financial incentives, there would be a mechanism by which partners could be held accountable for their level of participation (being less dependent on organizational priorities) | Accountability – incentive design |
| Be aware that the type of leadership can have influence on the commitment of the partners | Research projects for a healthy living environment. The national knowledge institute was the project leader. | 1. In two projects, the knowledge institute took the leading role at start. Also, by taking a leading role and not aligning with the needs and priorities of the partners. It was experienced as a project of the knowledge institute, not one ‘of the partners’ project’.  2. The national partner showed to be open for the needs of the local context (by having visits, and adjusting the project to local needs) which created acceptation for the project with the local partners | 1. The project partners did not always actively participate, as it was not always felt ‘their project’. The project leader experienced difficulties of sharing the accountability of certain tasks with other partners.  2. This created the feeling that the project was a collaboration addressing mutual needs, instead of a ‘research project’ | 1. Leadership – distributed leadership/Market – engagement  2. Market – local market structure |
| Communicating with the local residents during the project, by a representative or by providing updates helps in maintaining active participation | A project in which residents have a role in some executive tasks. The project is a research pilot in times of the corona pandemic. This caused some delays and made physical meetings with residents harder to organize. | 1. Due to delays in the process, the commitment of the residents to participate was experienced to decrease (according to one of the project partners)  2. Having a representative of the residents in the project group, who communicates about the project towards the local residents, ensured them to feel motivated to participate. | 1. This made residents feel less involved in the executive tasks in the project  2. This ensured that most residents participated throughout the whole project. | 1. Market – Engagement  2. Leadership - representation |
| Commitment of the different levels within organizations (e.g. municipalities) | Executive municipal representatives were included in the project, the managers were not included. When developing through the project, new decisions needed to be made, which required the commitment of different managers within the municipality | As some of these managers were not familiar with the project, they had no urgency in the continuation of the project | This made them question their level of involvement (they did not understand the need for participating and felt like it was a project of the national knowledge institute) | Market - agreement on purpose |

**3. Defining roles, tasks, and other prerequisites at the start of the project helps in pursuing the project over time, but needs re-examination throughout the project**

| **Strategy** | **Context** | **Mechanism** | **Outcome** | **CAHN** |
| --- | --- | --- | --- | --- |
| Make agreements about everyone’s roles, and tasks | Having a project with different local, regional and national partners. At the start of the projects, agreements about roles and tasks were written down. During the collaboration the partners were developing relationships with each other. | Having the roles and tasks written down, provided a basis to call on each other when tasks were not fulfilled.  A facilitating factor was the relationships that developed through the process, which made ‘ discussions’ about whether one performed ones tasks and roles less heavy. | Partners feel able to call on each other when tasks aren’t performed as was agreed upon.  Additionally, by developing relationships, they grow in their understanding what can be expected from whom and who is responsible for what. | Market – heedfulness  Market – trust, reciprocity, respect |
| Investing in the necessary support for partners when performing their roles and tasks | Different contexts in which the project partners were in need of extra support for performing their roles, e.g.  1. A local project in which a regional health services representative has an official link with the municipality, but also had a role within the project that sometimes conflicted with the link with the municipality.  2. A partner of which both the executive and managerial representatives are (sometimes) present in the project group | 1. The national knowledge institute sometimes ‘took over’ the role of the regional health service, when the official role and project role towards the municipality were conflicting. This helped the municipal health service in maintaining its’ official role.  2. By clear communication between both representatives of the same organization, the executive representative knows where to commit to in the project meetings (when the other representative is not present). | 1. The official role of the partner is not in ‘danger’.  2. The executive representative prevents impairment of his role, by only committing to things that can be done. | Relations – heedfulness  Leadership – distributed leadership |
| Take into account the expectations society has of your role as an organization in the collaborative project | Different examples were provided of how societal expectations affected the valuation of the roles of project partners | The expected role of the partner, according to (local) society, influences how the partner and execution of the role in the partnership is perceived.  1. Having the national knowledge institute in the project can create urgency to participate by other partners  2. Local partners are expected to address the needs of local society, by sharing results or by choosing to participate in the projects (even when not suitable or necessary in the project collaboration itself). | 1. This perspective on the role of the national knowledge institute, provides the opportunity to also take a more leading role in the collaboration  2. These expectations can influence the behavior of the local partners, and can influence the way that local partners are perceived (positive or negative) by local society | Social forces – normative social forces |
| Link the right task to the person with the right function (that is also held responsible for it) | A partner representative has a background in research and is involved as such in the project, though the tasks for the projects shift towards more executive/ implementation tasks. This is the responsibility of another representative from the partner organization. | As the responsibility of the representative was not aligned with its tasks, it was deliberated how involved this person would be in these tasks (especially with a lack of hours to spend on these tasks) | There was less active participation for these executive tasks, compared to when this person would have been responsible for the tasks.(now it was the wrong function at the wrong position) | Accountability - incentive design |
| Align necessary financial resources at the start of the project | A pilot project of which the executive phases were not fully elaborated at the onset of the project. | As there was no clear idea about the execution phase, and thus little insight in what financial budgets were needed, the partner organization did not reserve budget for these phases. | The budget needed to be arranged later in the process, which cost the partners more time | Finance - financial strategy |
